# The influence of parallel curb parking on traffic capacity at an intersection

**DOI:** 10.1016/j.heliyon.2023.e23935

**Published:** 2023-12-21

**Authors:** Qi Zhao, Yu-xuan Zhan, Lili Zhang, Lichen Su, Li Wang

**Affiliations:** a Beijing Key Laboratory of Urban Intelligent Traffic Control Technology, North China University of Technology, Beijing 100144, China; b China Academy of Transportation Science, Beijing 100029, China; c The College of Information Engineering, Beijing Institute of Petrochemical Technology, Beijing 102617, China; d School of Automation Science and Electrical Engineering Beijing, Beihang University 100083, China

**Keywords:** Curb parking, Intersection, Traffic capacity, Traffic wave

## Abstract

In this paper, we propose a model based on traffic flow theory to quantify the influence of curb parking on traffic capacity at a road intersection. The model takes into account vehicles parked at the curb and those that perform parking maneuvers, under three different saturation scenarios at the intersection. We apply the model to three input data sets and compare the results with those obtained from a traffic simulation. The model calculation and traffic simulation errors (MAPE and RMSE) are within an acceptable range (smaller than 10 %), which shows the effectiveness of the proposed model.

## 1 Introduction

The rapid growth of the urban parking demand, especially in the urban central area, has led to an increase in parking problems. Curb parking can alleviate the situation, but vehicles parked at the curb and vehicles undertaking curb-parking maneuvers can hinder the circulation [1]. Hence, traffic capacity will be affected by curb parking, especially at signalized intersections. From now on, we will only assume parallel curb parking, since it is the most common type of parking on roads.

Several studies have previously been conducted to analyse curb parking's influence on traffic. In 1968, Webster developed a model exploring the impact of curb parking on vehicle travel delays. He built a curb parking simulation model improving its physical representation by means of a periodic scan simulation method [2]. In 1973, Yu used computer simulation to determine the influence of curb parking on road capacity and on vehicle travel delays. The effects of curb-parking maneuvers on approaching vehicles were determined using time delay and applied to the roadway's capacity [3]. In 2003, Zeng analyzed three aspects related to curb-parking: the effective width of the lane, the lateral clearance, and the impact on traffic when entering and leaving the parking lot. These authors also developed the correction coefficient of the road capacity impact model [4]. In 2005, Chen explored the impact of curb parking on the traffic flow status, when entering and leaving the parking lot [5]. Arnott and Inci used quantitative measures to set up the parking area and established the corresponding relationship between saturation flow and effective road width to reflect the effects caused by curb parking on traffic flow [6]. Guo et al. proposed a model to estimate travel time, taking into account the effects of curb parking [7]. Portilla et al. used queue theory to analyse the average link journey times under the influence of curb-parking maneuvers and badly parked vehicles [8]. In 2011, Ye and Chen analyzed traffic slowdown as a function of the distance between the parking area and the intersection [9].

The above-mentioned studies are mainly focused on the impact of curb parking on traffic along road sections. In recent years, an increasing amount of research related to the impact of curb parking on intersections and traffic capacity calculation formula has been conducted. Cao et al. found that the distance between the parking lots and the intersection was highly influential on traffic performance at the intersection [10]. In 2009, Chen and He analyzed the variation in delay time caused by the curb parking nearest to the intersection stop line, using the curb parking area of Tianjin Road in Nanjing as a case study [11]. Cao et al. estimated the reduction in service rate attributable to the delay from an upstream curb-parking maneuver at different locations and provided suggestions for avoiding this reduction [12]. Madushanka identified the effects parking manoeuvres (which is the number of parking movements in unit time, expressed as vehicles per hour) have on the stream speed and capacity due to curb parallel parking in designated parking bays [13]. Kurek showed the need to conduct further research on the impact of parking maneuvers on the capacity of intersections with traffic lights for road traffic conditions in Poland [14]. Although there are two effects related to curb parking that can affect the traffic, that is, vehicles parked in the parking lots and those performing curb-parking maneuvers, most of the studies are focused on only one of them. The present study analyzes the influence of curb parking on traffic at an intersection, considering both the vehicles parked at the curb and the curb-parking maneuver. We propose a model for the influence of curb parking on traffic capacity at an intersection by using traffic flow theory and verify the model by traffic simulation. It has the potential to offer insights into the broader problem of roadway congestion stemming from curb parking practices. By conducting comprehensive simulations across diverse settings, this study could provide valuable solutions for managing and alleviating parking-related issues on a larger scale.

## 2 Methodology

**Fig. 1:**
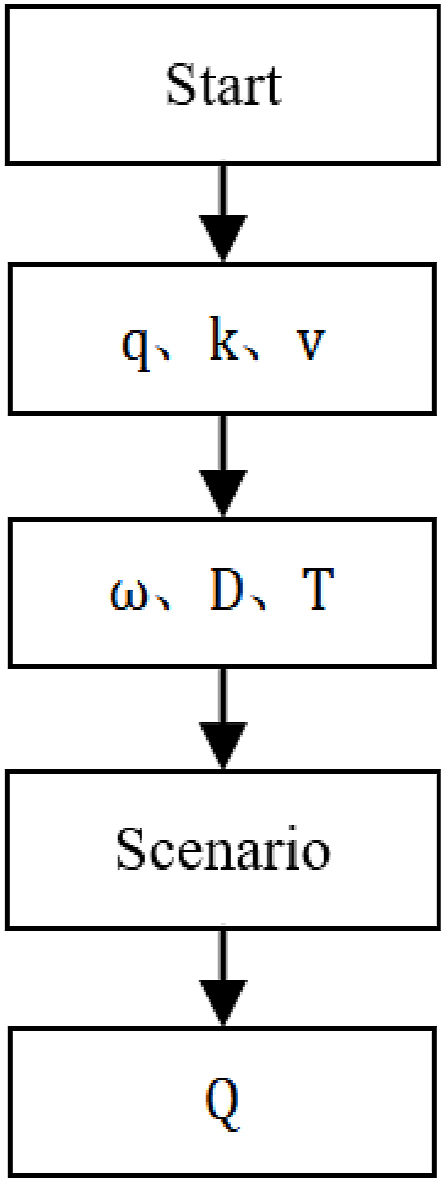
Flow chart for calculating traffic volume in different scenarios.

This study analyzes the impact of curb parking on the capacity at road intersection by using a model based on traffic flow theory. It is assumed that the road has only two lanes in two directions and thus all lanes are affected by curb parking. The model analyzes the two aspects by which the traffic capacity at the intersection can be affected by curb parking: the effect of vehicles parked at the curb and the effect of vehicles undertaking curb-parking maneuvers. To study the impact of curb parking on the capacity of an intersection, the model takes into account the duration of the curb-parking maneuver, the distance from the parking area to the intersection, and the volume of arriving traffic. The flow chart is shown in Fig. 1.

### 2.1 Effect of vehicles parked at the curb on traffic capacity at an intersection

**Fig. 2:**
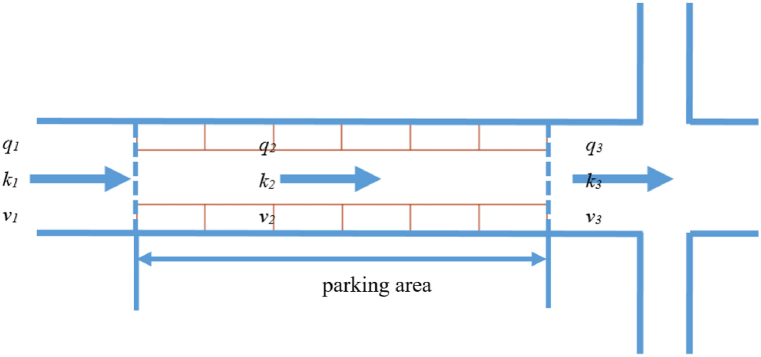
Traffic flow parameters in different road sections.

The road with curb parking is divided into three sections: upstream of parking area, parking area, and downstream of parking area. The traffic volume, traffic density, and average speed are defined in each of these three sections, and the parking area is 6 m long and 2.5 m wide, as shown in Fig. 2.

The traffic flow through the parking area produces traffic waves, and the process of traffic wave generation and dissipation is divided into the following three stages.

Stage 1: Once the traffic flow enters the parking area, the average speed will decrease owing to the influence of parked vehicles. The queuing wave is then generated and propagates upstream [15].
Stage 2: As the queuing wave propagates upstream, the vehicle continues to enter the parking area. After the traffic flow starts to exit the parking area, the start-up wave is generated and propagates upstream.
Stage 3: The start-up wave will meet the queuing wave when it propagates upstream and as a result the dissipation wave is generated. This means that the queue length reaches the maximum [16].

In these stages, the traffic flow parameters are as follows:

(1) $$\omega_{1}=\frac{q_{2}-q_{1}}{k_{2}-k_{1}}$$

where $\omega_{1}$ = speed of the queuing wave;

(2) $$\omega_{2}=\frac{q_{3}-q_{2}}{k_{3}-k_{2}}$$

where $\omega_{2}$ = speed of the start-up wave;

(3) $$T_{0}=\frac{L}{v_{2}}$$

where $T_{0}$ = time required for the vehicle to pass the parking area, L = length of the curb parking area and $v_{2}=$ average speed of the vehicle in the parking area;

(4) $$T_{1}=\frac{\omega_{2}{*T}_{0}}{\omega_{2}-\omega_{1}}$$

where $T_{1}$ = time until the queuing wave meets the start-up wave.

When the queuing wave meets the start-up wave, the queue length D reaches the maximum value:

(5) $$D=\omega_{1}*T_{1}$$

Based on the saturation condition, the following three scenarios are defined. Scenario 1 is that an upstream arrival vehicle can pass through the intersection in one signal cycle with or without curb parking. Scenario 2 is that an upstream arrival vehicle can pass through the intersection in one signal cycle without curb parking, but it cannot pass through the intersection in this signal cycle with curb parking. Scenario 3 is that an upstream arrival vehicle cannot pass through the intersection in this signal cycle with or without curb parking.

Scenario 1: The traffic at the intersection is under-saturated, so the vehicle can pass through the intersection in this signal cycle with or without curb parking.

Scenario 2: The intersection is under-saturated without curb parking but becomes over-saturated with curb parking.

Scenario 3: The intersection is over-saturated with or without curb parking.

Scenarios 1, 2, and 3 are set to have curb parking located in the exit lane of the intersection, i.e., on-street parking is located downstream of the intersection, namely (6).

(6) $$Q_{2}=\left\{ \begin{array}{c} 0,q_{1}\cdot c<S\cdot g-\left(\left(q_{1}-q_{2}\right)\cdot \left(T_{1}+\frac{D}{v_{3}}\right)\right)\\ q_{1}\cdot c-\left(S\cdot g-\left(\left(q_{1}-q_{2}\right)\cdot \left(T_{1}+\frac{D}{v_{2}}\right)\right)\cdot \frac{g}{c}\right),S\cdot g>q_{1}\cdot c>S\cdot g-\left(\left(q_{1}-q_{2}\right)\cdot \left(T_{1}+\frac{D}{v_{3}}\right)\right)\\ \left(q_{1}-q_{2}\right)\cdot \left(T_{1}+\frac{D}{v_{2}}\right)\cdot \frac{g}{c},q_{1}\cdot c>S\cdot g \end{array}\right.$$

where $Q_{2}$ = loss of capacity caused by vehicles parked at the curb, c = cycle length, g = green time for the direction of interest and S = saturation flow rate.

### 2.2 Effect of curb-parking maneuvers on traffic capacity at an intersection

Based on the saturation condition, the following three additional scenarios are defined. Scenario 4 is that an upstream arrival vehicle can pass through the intersection in this signal cycle with or without curb parking. Scenario 5 is that an upstream arrival vehicle can pass through the intersection in this cycle without curb parking or that an upstream arrival vehicle cannot pass through the intersection in this signal cycle with curb parking. Scenario 6 is that an upstream arrival vehicle cannot pass through the intersection in this cycle with or without curb parking. Similar to Scenarios 1, 2, and 3, Scenarios 4, 5, and 6 are set to have curb parking located in the inlet lane of the intersection, i.e., on-street parking is located upstream of the intersection, based on the saturation condition.

These scenarios are shown in Table 1.

**Table 1:** Different scenarios.

Scenario distribution		Degrees of traffic saturation	Time that the last vehicle before the parking area crosses the intersection
Scenario 4		$q_{1}\cdot c<S\cdot g-S\cdot p$	$T+T_{a}$ does not affect the traffic capacity at intersection
Scenario 5	Scenario 5.1	$S\cdot g>q_{1}\cdot c>S\cdot g-S\cdot p$	$T+T_{a}>c-g$
	Scenario 5.2		$T+T_{a}<c-g$
Scenario 6	Scenario 6.1	$q_{1}\cdot c>S\cdot g$	$T+T_{a}>c-g$
	Scenario 6.2		$T+T_{a}<c-g$

Scenario 4: The traffic at the intersection is under-saturated, so the traffic delay caused by curb-parking maneuvers will dissipate before the start of the next cycle. This means that the loss of capacity caused by curb-parking maneuvers is $Q_{1}=0$.

**Fig. 3:**
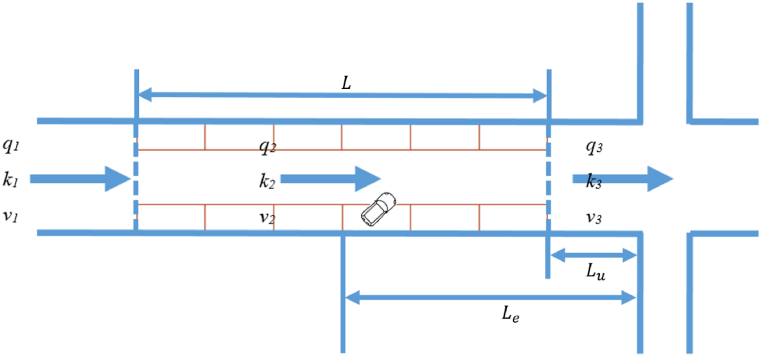
Schematic diagram of $L_{u}$, $L_{e}$ and L.

Scenario 5: The traffic at the intersection is under-saturated without the presence of curb-parking maneuvers but becomes over-saturated with curb-parking maneuvers. The two scenarios within Scenario 5 are described as follows. Scenario 5.1 is when the last vehicle before the parking area crosses the intersection during a green signal phase, and $T+T_{a}>c-g$. Scenario 5.2 is when the last vehicle before the parking area crosses the intersection during a red signal phase, and $T+T_{a}<c-g$. For both scenarios, T is the moment in which the curb-parking maneuver begins, p is the duration of the curb-parking maneuver, and $T_{a}$ is the travel time from the parking area of the curb-parking maneuver to the intersection(Equation (7)):

(7) $$T_{a}=\frac{L_{e}-L_{u}}{v_{2}}+\frac{L_{u}}{v_{3}}$$

where $L_{u}$ = distance between the curb parking area and the intersection, $L_{e}$ = distance between the parking area that has curb-parking maneuvers and the intersection, with $L_{e}\in \left(L_{u},L_{u}+L\right)$. Fig. 3 shows the schematic diagram of $L_{u}$, $L_{e}$ and L.

**Fig. 4:**
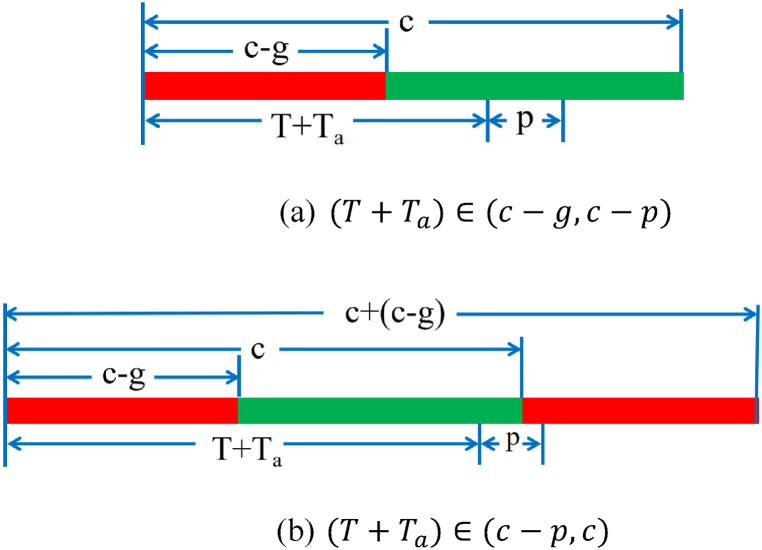
Two scenarios in Scenario 5.1.

Scenario 5.1: Two scenarios are defined for the time in which the last vehicle before the parking area crosses the intersection on a green time.When $\left(T+T_{a}\right)\in \left(c-g,c-p\right)$ is the time in which the first vehicle behind the parking area and the last vehicle before the parking area cross the intersection on a green time shown in Fig. 4 (a).

When $\left(T+T_{a}\right)\in \left(c-p,c\right)$ is the time in which the first vehicle behind the parking area crosses the intersection after the start of the next cycle, as shown in Fig. 4 (b).

The expression for the effect of curb-parking maneuvers on traffic capacity at an intersection is as Equation (8):

(8) $$Q_{1}=\left\{ \begin{array}{c} q_{1}\cdot c-S\cdot \left(g-p\right)\;\left(T+T_{a}\right)\in \left(c-g,c-p\right)\\ q_{1}\cdot c-\left(S\cdot g-S\cdot \left(c-\left(T+T_{a}\right)\right)\right)\;\left(T+T_{a}\right)\in \left(c-p,c\right) \end{array}\right.$$

where $Q_{1}$ = loss of capacity caused by the curb-parking maneuver.

**Fig. 5:**
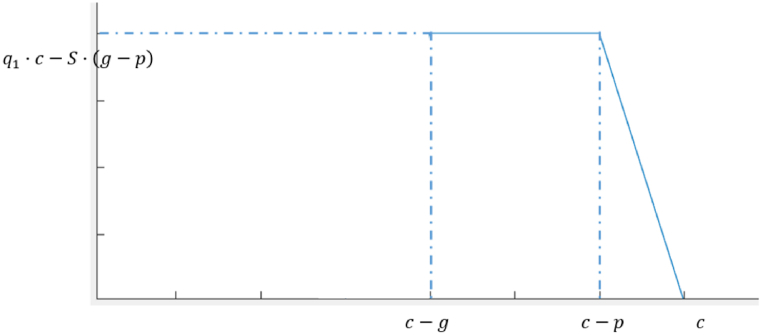
Reduction in traffic capacity in Scenario 5.1.

The relationship between cycle length and loss of capacity caused by the curb-parking maneuver is shown in Fig. 5, when $\left(T+T_{a}\right)\in \left(c-g,c-p\right)$, all the impact caused by the curb-parking maneuver is during the green time, and the effect of the curb-parking maneuver on traffic capacity at the intersection is at its maximum. When $\left(T+T_{a}\right)\in \left(c-p,c\right)$, as $T+T_{a}$ increases, the curb-parking maneuver time is shorter in the current cycle, and the loss of capacity caused by the curb-parking maneuver is less.

**Fig. 6:**
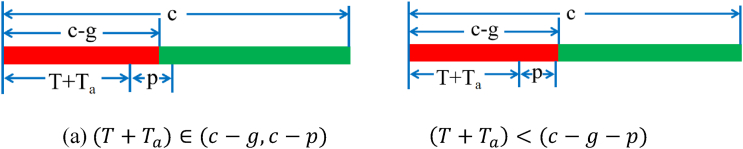
Two scenarios in Scenario 5.2.

Scenario 5.2: At the time in which the last vehicle before the parking area crosses the intersection on the red time, the following two scenarios are defined:

When $\left(T+T_{a}\right)\in \left(c-g-p,c-g\right)$ is the time in which the first vehicle behind the parking area crosses the intersection on a green time and the time in which the last vehicle before the parking area crosses the intersection on a red time shown in Fig. 6 (a).

When $\left(T+T_{a}\right)<\left(c-g-p\right)$ is the time in which the first vehicle behind the parking area and the last vehicle before the parking area cross the intersection on a red time shown in Fig. 6 (b).

The schematic diagram of the time of the first vehicle behind the parking area crosses the intersection on a red time is shown in Fig. 6.

The expression for the effect of curb-parking maneuvers on traffic capacity at an intersection is as Equation (9):

(9) $$Q_{1}=\left\{ \begin{array}{c} q_{1}\cdot c-S\cdot \left(g-(T+T_{a}+p-\left(c-g\right)\right)\;\left(T+T_{a}\right)\in \left(c-g-p,c-g\right)\\ 0\;\left(T+T_{a}\right)<\left(c-g-p\right) \end{array}\right.$$

Scenario 6: The intersection is over-saturated with or without curb-parking maneuvers. The two scenarios within Scenario 6 are as follows. Scenario 6.1 is that the time in which the last vehicle before the parking area crosses the intersection on a green time is $T+T_{a}>c-g$. Scenario 6.2 is that the time in which the last vehicle before the parking area crosses the intersection on a red time is $T+T_{a}<c-g$.

Scenario 6.1: At the time in which the last vehicle before the parking area crosses the intersection on a green time, the following two scenarios (as shown in Fig. 5) are defined:

When $\left(T+T_{a}\right)\in \left(c-g,c-p\right)$ , the last vehicle before the parking area and the last vehicle before the parking area cross the intersection on a green time.

When $\left(T+T_{a}\right)\in \left(c-p,c\right)$ is the time in which the first vehicle behind the parking area crosses the intersection after the start of the next cycle(Equation (10)).

(10) $$Q_{1}=\left\{ \begin{array}{c} S\cdot p\;\left(T+T_{a}\right)\in \left(c-g,c-p\right)\\ S\cdot \left(c-T-T_{a}\right)\;\left(T+T_{a}\right)\in \left(c-p,c\right) \end{array}\right.$$

**Fig. 7:**
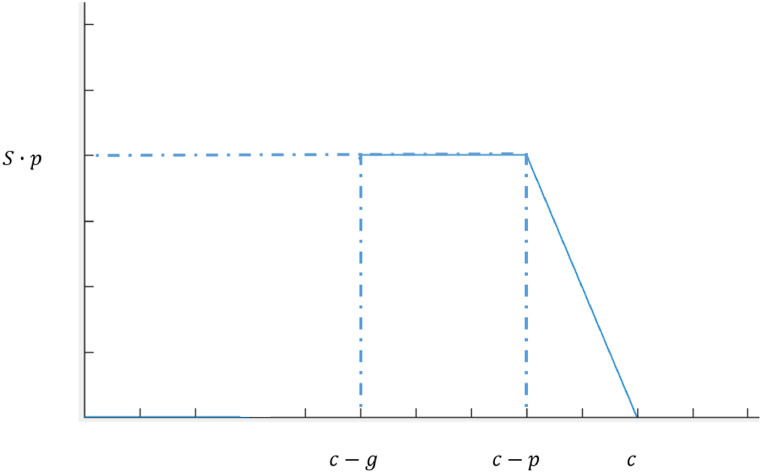
Reduction in traffic capacity in Scenario 6.1.

As shown in Fig. 7, the intersection is over-saturated both with or without curb-parking maneuvers and the analysis is similar to that of Fig. 3. When $\left(T+T_{a}\right)\in \left(c-g,c-p\right)$, all the impact caused by curb-parking maneuvers is at the green time, and the effect of curb-parking maneuvers on the traffic capacity at the intersection is at its maximum. When $\left(T+T_{a}\right)\in \left(c-p,c\right)$, as $T+T_{a}$ increases, the curb-parking maneuver time is shorter in the current cycle, and the loss of capacity caused by curb-parking maneuvers is less.

Scenario 6.2: At the time in which the last vehicle before the parking area crosses the intersection on a red time, the following two scenarios are defined: when $\left(T+T_{a}\right)\in \left(c-g-p,c-g\right)$ is the time in which the first vehicle behind the parking area crosses the intersection on a green time and the last vehicle before the parking area crosses the intersection on a red time; and when $\left(T+T_{a}\right)<\left(c-g-p\right)$ is the time in which the first vehicle behind the parking area and the last vehicle before the parking area cross the intersection on a red time(Equation (11)).

(11) $$Q_{1}=\left\{ \begin{array}{c} S\cdot \left((T+T_{a}+p-\left(c-g\right)\right)\;\left(T+T_{a}\right)\in \left(c-p-g,c-g\right)\\ 0\;\left(T+T_{a}\right)<\left(c-g-p\right) \end{array}\right.$$

### 2.3 Total effect on traffic capacity at an intersection

Combining the effects of curb-parking maneuvers and vehicles parked at the curb, the total impact of curb parking on traffic capacity at an intersection can be expressed in the following expression(Equation (12)).

(12) $$Q_{m}=\left\{ \begin{array}{c} 0\;S\cdot g-Q_{m}>\;q_{1}\cdot c\\ Q_{2}+\left(M\cdot P\cdot \left(Q_{1}-q_{1}\cdot c+S\cdot g\right)\right)\;S\cdot g-Q_{m}\;<\;q_{1}\cdot c<\;S\cdot g\\ Q_{2}+\left(M\cdot P\cdot Q_{1}\right)\;q_{1}\cdot c<\;S\cdot g \end{array}\right.$$

where $Q_{m}$ = loss of capacity caused by total curb parking, M = number of parking areas on the road and P = probability of a curb-parking maneuver.

According to the above equation, the loss of capacity caused by total curb parking is affected by the distance of the curb parking area to the intersection and by the length of the curb parking area. The authors of the present study are focused on the loss of capacity caused by the total curb parking effect due to the length of the curb parking area. Parameters such as the number of parking areas on the road and the duration of the curb-parking maneuver are defined as constants.

(1) Loss of capacity caused by total curb parking effect due to the distance between the curb parking area and the intersection.

The effect of curb-parking maneuvers on traffic capacity at an intersection is closely related to the distance $L_{u}$ between the curb parking area and the intersection: the range of $L_{e}$ increases as $L_{u}$ increases, and $T_{a}$ increases as $L_{u}$ increases. At the time in which the first vehicle behind the parking area crosses the intersection on a green time, in cases where other parameters are unchanged, $Q_{1}$ decreases as $T+T_{a}$ increases, as shown in Fig. 3, Fig. 5. At the time in which the first vehicle behind the parking area crosses the intersection on a red time, in cases where other parameters are unchanged, $Q_{1}$ increases as $T+T_{a}$ increases.

(2) Loss of capacity caused by total curb parking effect due to the length of the curb parking area

The length of the curb parking area L is positively correlated with the loss of traffic capacity at the intersection caused by curb parking. Under normal conditions, the increase in the length L of the curb parking area also means that the number M of parking areas increases. Moreover, M is positively correlated with traffic capacity at the intersection caused by curb parking.

### 2.4 Model simulation and Validation

#### 2.4.1 Model simulation

**Fig. 8:**
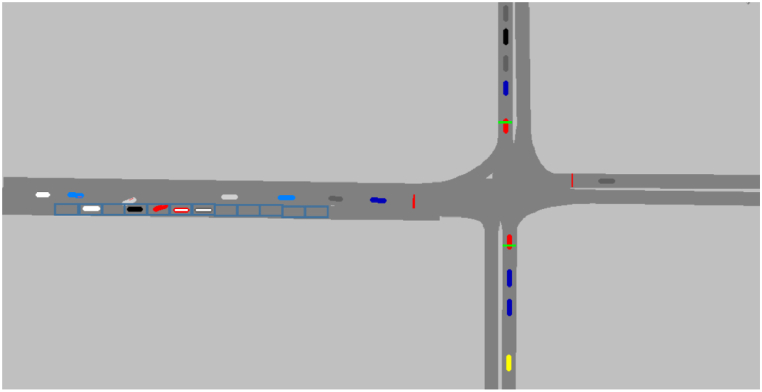
Vissim simulation Scenario.

To verify the model presented above, the traffic is simulated by assuming that the road has two lanes in two directions without a separated area with VISSIM simulation software. The intersection is shown in Fig. 8.

The data inputs of VISSIM are shown in Table 2:

**Table 2:** Data inputs to run the VISSIM.

Name of data inputs	Data 1	Data 2	Data 3	Data 4	Data 5
Flow rate	600 pcu/h	1200 pcu/h	1200 pcu/h	1500 pcu/h	1800 pcu/h
Speed before the vehicle enters the parking area	50 km/h	50 km/h	50 km/h	50 km/h	50 km/h
Duration of the curb-parking maneuver	6 s	6 s	6 s	6 s	6 s
Cycle length	60 s	60 s	60 s	80 s	80 s
Green time of the direction of interest	30 s	30 s	30 s	30 s	40 s
Number of parking areas on the road	12	12	12	12	12
Probability of a curb-parking maneuver	1/12	1/4	1/12	1/6	1/4

Data 1 and Data 3, Data 2 and Data 3 are controls. Data 4 and Data 5 are not controls variables and are meant to simulate the accuracy of the formula with multiple data changes.

The flow rates listed in Table 2 are all over-saturated flows, because they can reflect the loss of capacity. In addition to the data that appear in Table 2, there are other data not shown in it, corresponding to VISSIM's default settings.

#### 2.4.2 Model Validation

Taking data 2 in Table 2 as an example, the simulation results of VISSIM indicate that the flow rate is 1073.5 pcu/h without curb parking, and 999.6 pcu/h with curb parking. These are the average result obtained from 10 simulations with different random seeds. The loss rate after including the parking area is 73.9 pcu/h. On the other hand, the calculation results are as follows:

In accordance with Equations (10), (11), (12), the impact of curb-parking maneuvers on traffic capacity at an intersection is $E\left(Q_{1}\right)=$ 0.895, and M = 12, P = 1/12, thus $M\cdot P\cdot Q_{1}$ = 0.895 in a signal cycle. The number of signal cycles is 60 and $M\cdot P\cdot Q_{1}$ is 53.675 pcu/h.

Taking into account Equations (1), (2), (3), (4), (5)) and the data in Table 2, the calculation of the impact of the effect of vehicles parked at the curb on traffic capacity at the intersection is as follows:

$$\omega_{1}=1.04\;\text{km}/h$$

$$\omega_{2}=1.14\;\text{km}/h$$

$$T_{0}=3.9\;s=0.002\;h$$

$$T_{1}=\frac{\omega_{2}{*T}_{0}}{\omega_{2}-\omega_{1}}=28.8\;s=0.02\;h$$

$$D=\omega_{1}*T_{1}=24.2\;m=0.02\;\text{km}$$

In accordance with Equation 6, signal cycles, $Q_{2}$ = 14.76pcu/h.

Taking into account Equation (12), ${Q_{m}=Q}_{2}+\left(M\cdot P\cdot Q_{1}\right)$ = 68.43 pcu/h.

The results of the three sets of data in Table 2 are as follows, where the simulations are the average values of the simulation results of different random seeds for 10 consecutive times, as shown in Table 3.

(13) $$\text{RMSE}=\sqrt{\frac{\sum {\left(Q_{0}-Q_{m}\right)}^{2}}{n}}=6.62$$

(14) $$\text{MAPE}=\frac{\sum \frac{\left|Q_{0}-Q_{m}\right|}{Q_{0}}}{n}\times 100\%=5.41\%$$

where $Q_{0}$ = loss rate after adding parking area in VISSIM.

**Table 3:** Data Comparison with calculations and simulations.

Name of data inputs	Flow rate from upstream (pcu/h)	Loss rate after adding parking area in VISSIM (pcu/h)	$Q_{m\;}$ (pcu/h)	Difference between simulation and calculation
Data 1	597.2	7.7	0	/
Data 2	1073.5	73.9	68.43	7.4 %
Data 3	1073.5	182.74	175.79	3.8 %
Data 4	808.1	60.4	56.56	6.36 %
Data 5	1048.7	117.9	122.71	−4.08 %

To determine the overall difference between simulation and calculation, we calculate the root-mean-square error (RMSE) and the mean absolute percentage error (MAPE) as (13) and (14).

The values of RMSE and MAPE show that the overall difference is within an acceptable range(Because $Q_{m\;}$ = 0 calculated based on Data 1, The value of $\frac{\left|Q_{0}-Q_{m}\right|}{Q_{0}}$ does not change with the difference between $Q_{m\;}$ and Loss rate after adding parking area in VISSIM, which cannot play a role in determining the error. The data of Data 1 is not added in the calculation of MAPE).

The RMSE is used to measure the average size of the error, it is the square root of the average of the squared difference between the predicted value by using the proposed model and the simulation data by using traffic simulation. And the MAPE is used to measure the relative errors between the average test value and the real value on the test set. The closer the values of RMSE and MAPE are to 0 means the smaller the error between the simulated and real values. The results show that the model performs well in terms of fitting to the real data. The results show that the model performs well in terms of fitting to the real data.

## 3 Conclusion and Perspective

This study proposes a model to analyse the influence of curb parking and the curb-parking manoeuvre on traffic capacity at an intersection and it is based on traffic flow theory. The errors between model calculation and traffic simulation (RMSE and MAPE) are within an acceptable range, which shows the effectiveness of the proposed model.

It is concluded that the influence caused by curb parking, the influence caused by $Q_{1}$ is significantly higher than that caused by $Q_{2}$, and the loss of traffic capacity at an intersection increases as the curb parking area length increases. The width of curb parking areas will also have the influence on traffic, because it occupies the road space. However, the parking areas studied in this paper is the current prescribed style, so it is not considered as a variable.

Therefore, it is necessary to find a balance between parking and mobility. The results of this study can provide a theoretical basis for this equilibrium state. By adjusting the way parking spaces are set up, the possibility of traffic accidents caused by motor vehicles starting and driving from parking spaces is reduced; it also reduces the queuing time of vehicles, improves traffic congestion in local areas, and can also effectively improve the overall traffic operation of the road network, which has good application value. But in analyzing the impacts of curb parking road network capacity, this study only focuses on urban roadway. This results analyzes the impact of curb parking on the capacity of the road network and only influence the parallel curb parking lots which are more common in urban roads, while the impact of curb parking on the capacity of other road forms and curb parking areas needs to be further researched.

## Data availability

The data of “The Influence of Parallel Curb Parking on Traffic Capacity at an Intersection” that submitted to the “Heliyon” includes static data (parking space design standard) and dynamic data which is simulation experiment data.

Parking space design aspects of the data from the actual investigation and the parking space design related national standards, relevant documents can be downloaded on the https://jtgl.beijing.gov.cn/; Dynamic data is based on simulation Settings. This paper uses VISSIM to perform a simulation based on the data input of Table 2. The results used in this paper are based on the average value of the output results of 10 different random seeds.All data generated or analyzed during this study are included in this published article.

## Additional information

No additional information is available for this paper.

## CRediT authorship contribution statement

**Qi Zhao:** Writing – review & editing, Methodology, Data curation, Conceptualization. **Yu-xuan Zhan:** Writing – original draft, Project administration, Investigation, Formal analysis. **Lili Zhang:** Validation, Resources. **Lichen Su:** Visualization. **Li Wang:** Software, Investigation.

## Declaration of competing interest

The authors declare that they have no known competing financial interests or personal relationships that could have appeared to influence the work reported in this paper.

## Figures and Tables

**Table:** 

Name of notations	Meaning of notations
$v_{1},v_{2},v_{3}$	Vehicle average speed in the upstream of parking area, parking area, and downstream of parking area
${q_{1},q}_{2},q_{3}$	Traffic volume in the upstream of parking area, parking area, and downstream of parking area
$k_{1},k_{2},k_{3}$	Traffic density in the upstream of parking area, parking area, and downstream of parking area
$\omega_{1}$	Speed of the queuing wave
$\omega_{2}$	Speed of the start-up wave
$T_{0}$	Time required for the vehicle to pass the parking area
L	Length of the curb parking area
*D*	Queue length
$Q_{2}$	Loss of capacity caused by vehicles parked at the curb
c	Cycle length
g	Green time for the direction of interest
S	Saturation flow rate
$Q_{1}$	The loss of capacity caused by curb-parking maneuvers
T	The moment in which the curb-parking maneuver begins
p	The duration of the curb-parking maneuver
$T_{a}$	The travel time from the parking area of the curb-parking maneuver to the intersection
$Q_{m}$	Loss of capacity caused by total curb parking
M	Number of parking areas on the road and
P	Probability of a curb-parking maneuver
$Q_{0}$	Loss rate after adding parking area in VISSIM
